# Granulomatosis with polyangiitis with cardiac and large vessel involvement: a case report with a constellation of rare complications

**DOI:** 10.1093/ehjcr/ytae657

**Published:** 2024-12-14

**Authors:** Javaid Ahmad Dar, Vinod Nayanegali, Anand Manickavasagam, David Chase

**Affiliations:** Department of Cardiology, Christian Medical College, New Arcot Road, Vellore 632517, India; Department of Cardiology, Christian Medical College, New Arcot Road, Vellore 632517, India; Department of Cardiology, Christian Medical College, New Arcot Road, Vellore 632517, India; Department of Cardiology, Christian Medical College, New Arcot Road, Vellore 632517, India

**Keywords:** Complete heart block, Granulomatosis with polyangiitis, Aortitis, Sarcoidosis, Takayasu arteritis, Takotsubo cardiomyopathy, Pacemaker, Case report

## Abstract

**Background:**

Granulomatosis with polyangiitis (GPA) is an autoimmune multisystem disorder characterized by small vessel vasculitis with granulomatous inflammation. In this report, we describe a unique case of GPA who presented with complete heart block (CHB) and developed complications due to intracranial large vessel involvement.

**Case summary:**

A 47-year-old gentleman presented with CHB with a background history of arthralgia and blood-tinged nasal discharge. Whole body positron emission tomography–computed tomography scan showed soft tissue thickening with increased fluorodeoxyglucose uptake in basal interventricular septum and mitral leaflet aorta from the root up to the renal arteries. The patient developed subarachnoid haemorrhage and stress-induced cardiomyopathy after pacemaker implantation. The patient responded dramatically to steroids and rituximab and the CHB resolved on follow-up.

**Discussion:**

Cardiac involvement in GPA is very rare as is the large vessel involvement. In this report, we describe the cardiac involvement of GPA in the form of basal interventricular septum and anterior mitral leaflet giving rise to CHB. The patient also had aortitis and vertebral artery aneurysm, which ruptured resulting in subarachnoid haemorrhage. The patient also developed stress-induced cardiomyopathy and monomorphic ventricular tachycardia. The patient improved with steroids and rituximab and is doing well on follow-up.

**Learning points**

- Granulomatosis with polyangiitis is one of the rare causes of reversible complete heart block.
- Granulomatosis with polyangiitis can lead to inflammation of the anterior mitral leaflet and the basal interventricular septum and mimic cardiac sarcoidosis.
- Granulomatosis with polyangiitis can rarely involve large vessel arteries and lead to aneurysm formation.

## Introduction

Granulomatosis with polyangiitis (GPA) is an autoimmune multisystem disorder characterized by small vessel vasculitis with granulomatous inflammation.^1^ The pathological *sine qua non* for the diagnosis of GPA is an upper and lower respiratory tract with kidney involvement in the presence of characteristic immunological markers, i.e. autoantibodies against the protein, neutrophil proteinase-3, also called anti-neutrophilic cytoplasmic antibody. The cardiac involvement *per se* is very rare in GPA as are the large vessel aneurysms. In this article, we describe a unique case of GPA who presented with complete heart block (CHB) and developed complications due to intracranial large vessel involvement.

## Summary figure

**Figure:**
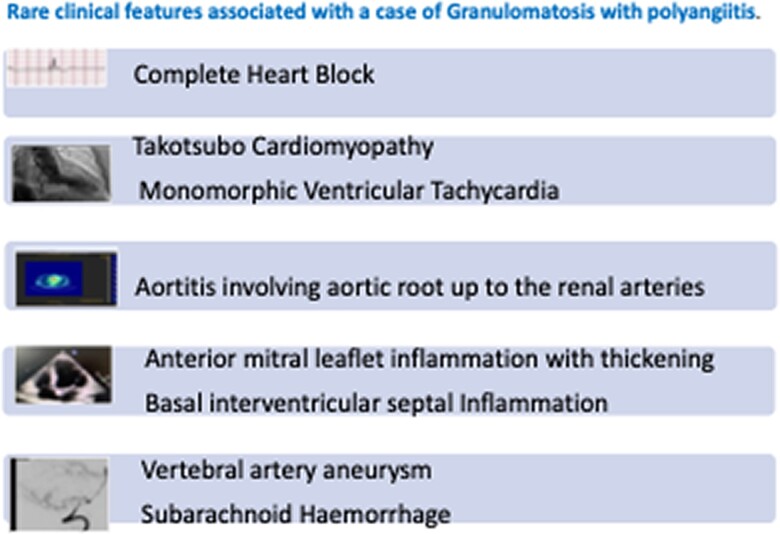


## Case presentation

**Figure 1:**
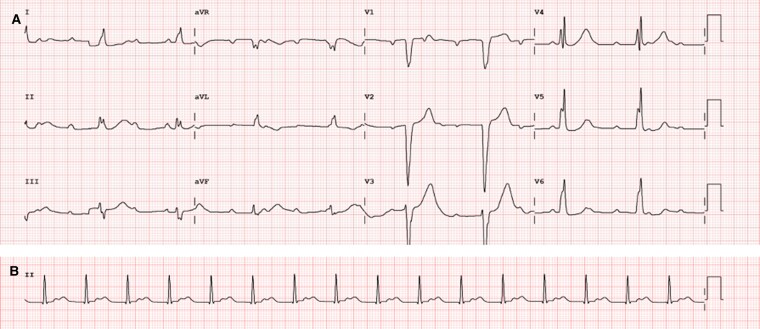
(*A*) Electrocardiogram at presentation showing complete heart block. (*B*) An electrocardiogram done a few days before the presentation showing a very prolonged PR interval (400 ms) with 1:1 atrioventricular conduction.

**Figure 2:**
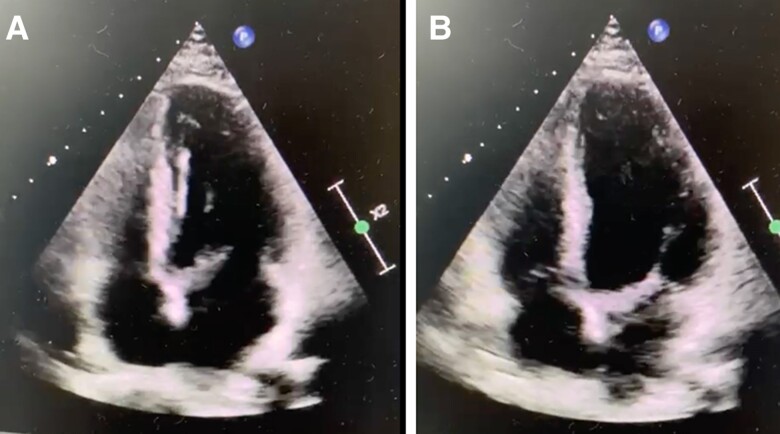
Echocardiographic images in a diastolic frame showing a thickened anterior mitral leaflet in diastole (*A*) and on the right side a systolic frame (*B*).

**Figure 3:**
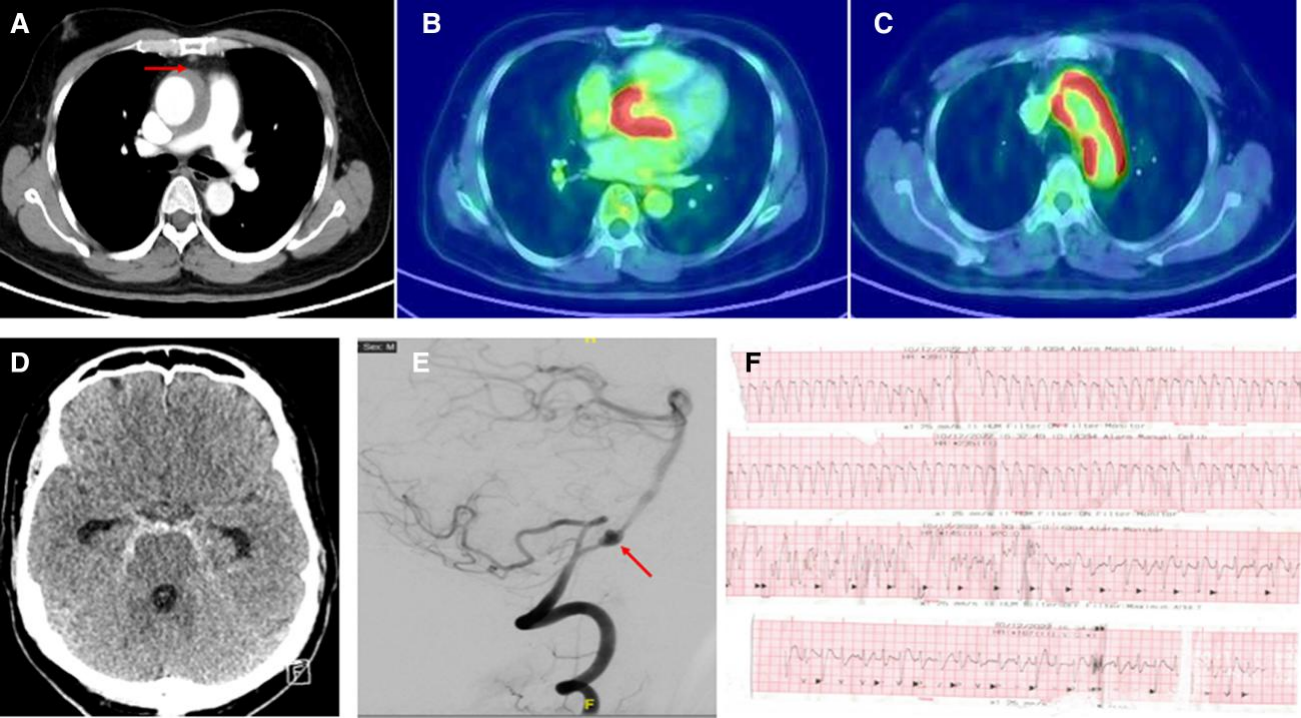
(*A*) A computed tomography section at the level of pulmonary artery bifurcation showing soft tissue thickening around the ascending aorta (see the arrow). In (*B*), the cardiac computed tomography section in the cardiac long axis shows fluorodeoxyglucose uptake in the basal interventricular septum and over the anterior mitral leaflet. In (*C*), the arch of the aorta shows avid fluorodeoxyglucose in the aortic wall. Computed tomography scan as shown in (*D*) shows subarachnoid haemorrhage with hyperintense cisterns. (*E*) A computed tomography angiogram of the right posterior circulation with a large aneurysm (see the arrow) in the vertebral artery. (*F*) Electrocardiogram shows monomorphic ventricular tachycardia.

A 47-year-old gentleman presented with a history of fatigue, bilateral wrist arthralgia, and blood-tinged nasal discharge 6 months ago. He was a non-smoker and had no history of any substance abuse. He had no significant past medical history. On examination, he was found to have a heart rate of 40 b.p.m. and blood pressure of 120/70, with normal systemic examination. There was no evidence of any sinusitis or arthritis or any other significant medical history. An electrocardiogram (ECG) revealed a CHB with ventricular escape rhythm at the rate of 50 b.p.m. (*Figure 1A*). One more ECG that the patient had done a few days before the current presentation revealed prolonged PR with 1:1 atrioventricular (AV) conduction (*Figure 1B*). Echocardiography was done, which revealed normal left ventricular (LV) function and normal valvular function; however, the anterior mitral leaflet was found to be thickened with no vegetations (*Figure 2*). Chest X-ray was normal; however, his computed tomography (CT) scan showed few nodules in the left upper lobe. Complete blood count was remarkable for thrombocytosis with a platelet count of 5.7 × 10^3^/mm^3^. Erythrocyte sedimentation rate was raised to 50 mm in the first hour and the C-reactive protein was raised to 7 mg/dL. Kidney function and liver function tests were normal; however, urine examination revealed proteinuria with no active sediment. The 24-h urinary protein revealed microalbuminuria of 330 µg of proteins (normal range <30 mg/24 h). Serum antinuclear antibody assay was normal; however, c-ANCA was positive (>200 IU/mL, normal range 0.00–0.22 units/mL). A whole body positron emission tomography (PET) scan was done for the patient in view of suspicion of sarcoidosis vs. vasculitis with unusual clinical features, which revealed thickened mitral leaflet with active fluorodeoxyglucose (FDG) uptake, which involved the ascending aorta, the arch, and the descending aorta up to the renal arteries suggestive of aortitis (*Figure 3A–C*). A diagnosis of GPA was made, and a plan for immuno-suppressive therapy after pacing was made. The pacemaker was implanted without any complications; however, on the second day of the pacemaker implantation, the patient developed an episode of seizure followed by an episode of monomorphic ventricular tachycardia for which he was cardioverted by direct current cardioversion. The patient became drowsy after these episodes and had to be managed with endotracheal intubation and mechanical ventilation. At this point, his serum electrolytes were normal and the CT scan of the brain revealed subarachnoid haemorrhage (SAH) (*Figure 3D*). Computed tomography angiography of the brain revealed a large aneurysm of the V4 segment of the right vertebral artery (*Figure 3E*). Repeat echocardiography showed severe LV dysfunction with LV apical ballooning consistent with stress-induced cardiomyopathy. Serum troponins were mildly raised; however, there was no serial elevation of the cardiac enzymes to suggest acute coronary syndrome. After managing the patient in neuro-critical care with a ventriculoperitoneal (VP) shunt for about a week, the patient improved and then underwent coil embolization of the vertebral artery aneurysm. Repeat echocardiographic examination showed normalization of the LV functions with no regional wall motion abnormalities. The patient was also started on oral steroids. After 3 weeks, the patient was discharged from the hospital in stable condition without any neuro-deficit. However, he presented again with headache and drowsiness after 5 days of the discharge and the repeat brain imaging revealed a large haematoma at the insertion of the VP shunt in the temporal lobe. The patient was managed conservatively with measures to reduce intracranial hypertension, and the serial brain imaging showed resolution of the haematoma. The patient was discharged again on steroids, ramipril, bisoprolol, amiodarone, and rituximab. Amiodarone was stopped on the follow-up after 6 months after the CT coronary angiography was done, which showed normal coronaries. Intracardiac defibrillator upgrade of the pacemaker was considered initially; however, in view of normalization of LV functions and the likely reversible aetiology of the VT, it was not pursued.

After 18 months of follow-up on steroid therapy, the mitral thickening resolved and the pacemaker interrogation showed good intrinsic rhythm with minimal ventricular pacing requirement (being <1%). There was normalization of the inflammatory markers, and the repeat serum ANCA serology was negative. Repeat PET scan was not done as it was not deemed to be clinically relevant.

## Discussion

Granulomatosis with polyangiitis is a small vessel vasculitis with systemic involvement and usually presents with renal and respiratory tract involvement.^2^ Cardiac involvement in GPA is rare and has been variably reported between 1% and 5%.^3^ Among the cardiac involvement, pericarditis is the most common pathology followed by valvular abnormalities, coronary arteritis, and conduction disorders. Complete heart block as the initial manifestation is very rare and has been reported in a handful of cases in the literature.^4,5^ Pacing was considered the safest approach in the management of this patient due to uncertain course of the disease. All cases of myocarditis other than a viral myocarditis like sarcoidosis and vasculitis warrant pacing as the natural course is quite variable and the heart block may not necessarily be reversible. After discussing with the patient, a safer strategy of pacing was chosen.

The valvular abnormalities in GPA are rare, although, in a series of patients with GPA, subclinical aortic regurgitation was reported to be much common.^6^

The peculiar abnormality in our case was a very thickened anterior mitral leaflet without any vegetations. Such inflamed leaflets are very rare; however, a case of GPA with thickened aortic and mitral leaflets with histologically confirmed granulomatous inflammation has been reported previously.^7^ Another rare clinical feature in our patient was aortitis as evidenced by thickening and the FDG uptake in PET scan in the aortic root, arch, and the descending aorta up to the renal arteries. The aortitis and renal involvement on CT scan simulated the radiological picture of Takayasu arteritis, which is a common cause of aortitis. However, the pathognomonic multi-organ involvement was highly unlikely for Takayasu arteritis. Large vessel aneurysms are a rare manifestation of GPA; however, all major arterial involvement has been reported including the aortitis and the aortic aneurysms.^8–10^ Our patient had a catastrophic clinical course due to the rupture of vertebral artery aneurysm and SAH, which was managed by coil embolization. Intracranial aneurysms are extremely rare and have been previously reported in one case from Japan in which immuno-suppressive therapy led to the disappearance of the aneurysms.^11^ Another novel cardiac involvement in our case was stress-induced cardiomyopathy, which was diagnosed after SAH. We believe this arose after SAH, which is a known trigger for stress-induced cardiomyopathy. The patient also developed monomorphic VT, which could have been due to the disease-related myocardial inflammation or due to stress-induced cardiomyopathy.^12^

In our case, the initial clues to the diagnosis were the longstanding symptoms of nasal discharge and arthralgia though the active arthritis at the time of presentation was absent. The diagnosis was confirmed by the multisystem involvement in the form of pulmonary nodules, microscopic haematuria with maxillary sinusitis in the presence of raised inflammatory markers, and high titres of c-ANCA. The patient after the initial catastrophic clinical course responded dramatically to immuno-suppressive therapy. The immuno-suppressive treatment strategy was discussed in a multidisciplinary team meeting. In view of life-threatening disease, treatment with second immuno-suppressive agent was recommended. For induction of remission in patients with new-onset or relapsing GPA with organ-threatening or life-threatening disease, the European Alliance of Associations for Rheumatology guidelines recommend treatment with a combination of glucocorticoids and either rituximab or cyclophosphamide, with rituximab being the preferred option in relapsing disease.^13^ After discussion with the patient, it was decided to combine rituximab with glucocorticoids. Cyclophosphamide was considered; however, the patient did not consent for it due to fertility issues.

The heart block and the thickening of the mitral leaflet reversed and the inflammatory markers showed full resolution of the inflammation. The long-term prognosis of GPA has markedly improved in recent years with the introduction of newer immunosuppressants.^14^

## Data Availability

The data underlying this article will be shared on reasonable request to the corresponding author.

## Lead author biography

The author has completed cardiology training from All India Institute of Medical Sciences, New Delhi in 2020 and then completed fellowship in electrophysiology in Christian Medical College, Vellore, India, in 2022. The author works at present as an assistant professor in the department of cardiology. The author has a keen interest in cardiac arrhythmias, heart failure, and sudden cardiac death.

## References

[1] Kronbichler A, Bajema IM, Bruchfeld A, Mastroianni Kirsztajn G, Stone JH. Diagnosis and management of ANCA-associated vasculitis. Lancet 2024;403:683–698. 38368016 10.1016/S0140-6736(23)01736-1

[2] Greco A, Marinelli C, Fusconi M, Macri GF, Gallo A, De Virgilio A, et al Clinic manifestations in granulomatosis with polyangiitis. Int J Immunopathol Pharmacol 2016;29:151–159. 26684637 10.1177/0394632015617063 PMC5806708

[3] McGeoch L, Carette S, Cuthbertson D, Hoffman GS, Khalidi N, Koening CL, et al Cardiac involvement in granulomatosis with polyangiitis. J Rheumatol 2015;42:1209–1212. 25934819 10.3899/jrheum.141513 PMC4505809

[4] Ghaussy NO, Du Clos TW, Ashley PA. Limited Wegener’s granulomatosis presenting with complete heart block. Scand J Rheumatol 2004;33:115–118. 15163113 10.1080/03009740310004063

[5] Valente F, Rozen L, Carlier S, Godart P. An uncommon case of complete AV block. BMC Cardiovasc Disord 2022;22:429. 36175842 10.1186/s12872-022-02866-5 PMC9520797

[6] Borowiec A, Rosinska M, Kowalik I, Rybski S, Chwyczko T, Jankowski J, et al Cardiac valvular involvement in granulomatosis with polyangiitis in long-term observation. Rev Port Cardiol 2024;43:97–103. 38122897 10.1016/j.repc.2023.08.008

[7] Espitia O, Droy L, Pattier S, Naudin F, Mugniot A, Cavailles A, et al A case of aortic and mitral valve involvement in granulomatosis with polyangiitis. Cardiovasc Pathol 2014;23:363–365. 25194969 10.1016/j.carpath.2014.07.007

[8] Blockmans D, Baeyens H, Van Loon R, Lauwers G, Bobbaers H. Periaortitis and aortic dissection due to Wegener’s granulomatosis. Clin Rheumatol 2000;19:161–164. 10791632 10.1007/s100670050038

[9] Shitrit D, Shitrit AB, Starobin D, Izbicki G, Belenky A, Kaufman N, et al Large vessel aneurysms in Wegener’s granulomatosis. J Vasc Surg 2002;36:856–858. 12368751 10.1067/mva.2002.126088

[10] Aoki N, Soma K, Owada T, Ishii H. Wegener’s granulomatosis complicated by arterial aneurysm. Intern Med Tokyo Jpn 1995;34:790–793. 10.2169/internalmedicine.34.790 8563123

[11] Takamasu E, Hattori S, Yokogawa N, Shimada K. Multiple aneurysms in granulomatosis with polyangiitis. J Rheumatol 2024;51:97. 10.3899/jrheum.2023-0510 37582553

[12] D’amico AT, Avenoso D, Ghiglieno C, Porcellini S, Dell’era G, Patti G. 48 a case of recurrent ventricular tachycardia in a patient with granulomatosis with polyangiitis with a challenging identification of arrhythmic substrate: the role of multimodal imaging. Eur Heart J Suppl 2022;24:suac121.034.

[13] Hellmich B, Sanchez-Alamo B, Schirmer JH, Berti A, Blockmans D, Cid MC, et al EULAR recommendations for the management of ANCA-associated vasculitis: 2022 update. Ann Rheum Dis 2024;83:30–47. 36927642 10.1136/ard-2022-223764

[14] Kumar A, Dembla G, Abrol A, Tiwari SC, Goel A, Bansal R. Clinical profile and long-term outcome of granulomatosis with polyangiitis (GPA): a corporate hospital-based study from northern India. Indian J Rheumatol 2015;10:183–188.

